# Risk Factors of Arteriovenous Fistula Stenosis in Hemodialysis Patients Using Electronic Stethoscope Monitoring: Prospective Pilot Cohort Study

**DOI:** 10.2196/97202

**Published:** 2026-08-18

**Authors:** Wen-Yi Li, Yi-Chen Wu, Bor-Wen Cheng, Feng-Jung Yang

**Affiliations:** 1 Division of Nephrology, Department of Internal Medicine, National Taiwan University Hospital, Yunlin Branch, Douliu, Taiwan; 2 National Taiwan University College of Medicine, Taipei, Taiwan; 3 Department of Industrial Engineering and Management, National Yunlin University of Science and Technology, Douliu, Taiwan; 4 Department of Medical Genetics, National Taiwan University Hospital, Children's Hospital Building, 8 Chung-Shan South Road, Taipei, 10041, Taiwan, 886-2-23123456; 5 Division of Nephrology, Department of Internal Medicine, National Taiwan University Hospital, Taipei, Taiwan

**Keywords:** arteriovenous fistula stenosis, hemodialysis, electronic stethoscope, acoustic monitoring, phonoangiography, vascular access surveillance, risk factors

## Abstract

**Background:**

Arteriovenous fistula (AVF) stenosis is an important complication of hemodialysis that may impair dialysis adequacy and patient outcomes. Conventional AVF monitoring relies largely on subjective physical assessment and may delay recognition of abnormal vascular access findings.

**Objective:**

This prospective pilot study aimed to assess the feasibility of serial electronic stethoscope monitoring for detecting abnormal AVF sounds before routine nursing assessment, and explore factors associated with a high-risk AVF phenotype, defined as percutaneous transluminal angioplasty within the preceding 3 years.

**Methods:**

We conducted a 3-month prospective study at the National Taiwan University Hospital Yunlin Branch Hemodialysis Center from March to June 2023. Thirty adults receiving maintenance hemodialysis through a functional AVF were enrolled; 29 completed follow-up and generated 1462 acoustic recordings. AVF sounds were recorded 2-6 times weekly at 2 standardized sites: approximately 3 cm proximal to the anastomosis and the arterial needle insertion site. Signals were analyzed using short-time Fourier transform and expressed as decibels relative to full scale. Clinical, demographic, laboratory, and dialysis-related data were collected. Because recordings were clustered within patients, risk-factor analyses were performed at the patient level. Associations with the prespecified high-risk AVF phenotype were explored using Fisher exact tests and Firth penalized logistic regression because only 9 patients met the high-risk definition.

**Results:**

An exploratory in-sample candidate threshold for abnormal AVF sounds was derived at <−28.59 decibels relative to full scale using a 3SD rule. Because the threshold was developed, refined, and evaluated within the same small dataset, it requires independent external validation. One participant had stenosis confirmed by duplex Doppler before acoustic monitoring began. In this illustrative case, the acoustic signal remained abnormal and was recognized approximately 1 month before routine bedside nursing assessment, although not before imaging. All stenotic recordings originated from this single participant and therefore do not constitute independent evidence of diagnostic performance. In exploratory patient-level analyses, the high-risk phenotype was associated with diabetes mellitus (odds ratio [OR] 12.6, 95% CI 2.2-138.1; *P*=.005), hypertension (OR 12.9, 95% CI 1.3-31.7; *P*=.033), and BMI ≤25 kg/m² (OR 19.0, 95% CI 2.0-2568; *P*=.011). Fresenius FX-series dialyzer use was not significant on exact testing (OR 4.4, 95% CI 0.9-28.5; *P*=.11). Estimates were imprecise because of the limited number of high-risk patients.

**Conclusions:**

Serial electronic stethoscope monitoring was feasible. In one illustrative patient with Doppler-confirmed stenosis, persistent acoustic abnormalities were recognized before routine nursing assessment but not before imaging. The candidate acoustic threshold and exploratory correlates of high-risk AVF status require confirmation in larger, externally validated prospective cohorts before clinical implementation.

## Introduction

End-stage renal disease (ESRD) represents a global health challenge with substantial impact on health care systems and patient quality of life. Taiwan exhibits the world’s highest ESRD prevalence at 3824 per million population in 2023, exceeding Japan (second highest at 2751 per million) by 39% [1]. The annual incidence of ESRD remains on an upward trend, reaching 12,459 new dialysis cases in 2022 with hemodialysis (HD) comprising 90.5% of all renal replacement therapies [2].

The arteriovenous fistula (AVF) serves as the lifeline for HD patients, providing reliable vascular access for thrice-weekly treatments [3]. National Kidney Foundation guidelines recommend AVF as the preferred vascular access due to superior patency rates and lower infection risk compared with arteriovenous grafts (AVGs) or central venous catheters [4]. However, AVF stenosis remains a critical complication, potentially causing inadequate dialysis, access thrombosis, and increased morbidity and mortality [5].

Current clinical surveillance of AVF relies predominantly on physical examinations performed by experienced nursing staff, who monitor for clinical indicators such as a diminished thrill, altered bruit characteristics, or pulsatile flow—all of which may suggest significant stenosis [6]. However, this subjective approach is constrained by several inherent limitations. First, the diagnostic accuracy is highly susceptible to interobserver variability, as the assessment depends heavily on the individual clinical expertise and the tactile or auditory acuity of the practitioner. Furthermore, physical examination often results in delayed detection, typically identifying stenosis only after it has progressed to hemodynamically significant narrowing. Finally, the qualitative nature of these subjective assessments precludes precise quantification, making it difficult to objectively track or compare fistula health longitudinally over time. While imaging modalities (Doppler ultrasound and angiography) provide definitive stenosis diagnosis, their use as routine screening tools is limited by cost, availability, and need for specialized personnel [7]. This gap between subjective clinical monitoring and expensive imaging creates an opportunity for intermediate surveillance technologies.

Electronic stethoscopes enable digital phonoangiography—the recording and quantitative analysis of vascular sounds—by facilitating objective measurement, storage, and analysis of AVF acoustic sounds [8]. Recent studies have demonstrated their potential in AVF monitoring. Wang et al [9] used short-time Fourier transform (STFT) analysis to differentiate stenotic from nonstenotic AVF based on frequency characteristics (stenosis: 600‐800 Hz vs nonstenosis: 200‐600 Hz). Tsuboi et al [10] found significant correlation between AVF sound intensity and Doppler ultrasound flow measurements, with 66%‐82% diagnostic accuracy for AVF dysfunction. Malindretos et al [11] demonstrated that dysfunctional AVFs exhibited mean amplitude <−40 decibel (dB) compared with normal fistulas at −20 dB. More recently, Presta et al [12] reported the feasibility of a new-generation digital stethoscope for AVF assessment—the closest comparator to the present approach—while Colombo et al [13] used a Doppler-derived blood flow or resistance index ratio (Qx) to predict stenosis and future thrombotic events, and Zhou et al [14] applied deep learning to blood flow sounds for automated stenosis detection. However, previous studies have some limitations, such as small sample sizes, cross-sectional designs, and lack of specific threshold values for stenosis detection in clinical practice.

In this prospective pilot study, we adopted an exploratory, multifaceted approach. First, we implemented serial longitudinal monitoring over a 3-month period to assess the feasibility of capturing temporal acoustic changes preceding clinical stenosis. Second, we derived a candidate decibel cutoff for abnormal AVF sounds, which we regard as exploratory rather than validated. Third, we examined whether electronic auscultation could flag abnormalities earlier than routine bedside nursing assessment. Finally, we explored, in a hypothesis-generating manner, patient factors associated with a high-risk AVF phenotype. We did not aim to establish a definitive diagnostic threshold or a confirmatory multivariable risk model.

## Methods

### Study Design and Population

This prospective observational cohort study was conducted at National Taiwan University Hospital (NTUH) Yunlin Branch Hemodialysis Center. The study protocol was approved by the Institutional Review Board of NTUH (IRB number 202302099RINA) and registered at ClinicalTrials.gov (NCT07436559). From March to June 2023, patients were screened for eligibility at the HD center. Individuals were eligible for inclusion if they were aged between 20 and 99 years, had been receiving maintenance HD for at least 3 months, possessed a functional native AVF, and demonstrated the capacity to provide written informed consent. Conversely, patients were excluded if they used an AVG or a tunneled cuffed central venous catheter (Permcath) for vascular access. Additionally, vulnerable populations who were unable to provide autonomous informed consent were excluded from the study.

The HD center provides care for a total of 156 patients, with a vascular access distribution comprising 124 (79.5%) patients with AVF, 21 (13.5%) patients with AVG, and 11 (7.0%) patients with Permcath. From this initial cohort, a total of 30 patients who met all eligibility criteria were enrolled in the study. During the follow-up period, 1 participant withdrew on May 22, 2023, citing personal reasons. Consequently, 29 patients completed the full study protocol and were included in the final analysis (Multimedia Appendix 1).

### Ethical Considerations

This study was approved by the Research Ethics Committee or IRB of NTUH (IRB number 202302099RINA) and was prospectively registered at ClinicalTrials.gov (NCT07436559). Written informed consent was obtained from every participant before enrollment. All acoustic recordings and clinical data were deidentified and stored under a study code; the linking key was held separately and was accessible only to the investigators, and no identifying information is presented in this report. Participants received no financial compensation for participation.

### Clinical and Laboratory Data Collection

At study entry, comprehensive demographic and clinical profiles were established for all participants. Baseline data included age, gender, and BMI, alongside a review of relevant comorbidities such as diabetes mellitus, hypertension, and dyslipidemia. Lifestyle factors, specifically smoking status, were also recorded. Regarding vascular access, we documented specific AVF characteristics, including its anatomical location (forearm vs upper arm), the duration since the creation of the access, and the patient’s overall dialysis vintage. Furthermore, we recorded detailed dialysis prescription parameters, encompassing dialyzer type and size (eg, F, FX [Fresenius FX-series], FLX [Fresenius FLX-series], and B3 series), dialysate composition, and the gauge of the needles used. The membrane materials of F, FX, and FLX were polysulfone, and B3 was polymethylmethacrylate. Anticoagulation protocols, specifically loading and maintenance heparin dosages, were also standardized and recorded. To account for previous vascular issues, we documented prior interventions, including the cumulative number of percutaneous transluminal angioplasties (PTAs) and the specific locations of any known stenoses. We defined the high-risk stenosis group as receiving PTA within 3 years and the stable patency group as not receiving PTA within 3 years. Pharmacological data, particularly the use of erythropoiesis-stimulating agents, were also collected.

To monitor the clinical status of the participants throughout the study period, laboratory and session-specific data were collected on a monthly basis. Serum albumin levels were obtained from routine monthly laboratory evaluations performed at the HD center as an indicator of nutritional status and chronic inflammation. Comprehensive data were extracted from dialysis machine records to characterize the hemodynamic environment of the AVF during treatment. These parameters included mean systolic blood pressure (BP); diastolic BP range (defined as the difference between the maximum and minimum diastolic BP); mean, initial, and maximum venous pressures; transmembrane pressure range; and the prescribed blood flow rate.

### Signal Processing and Acoustic Analysis

**Figure 1.**
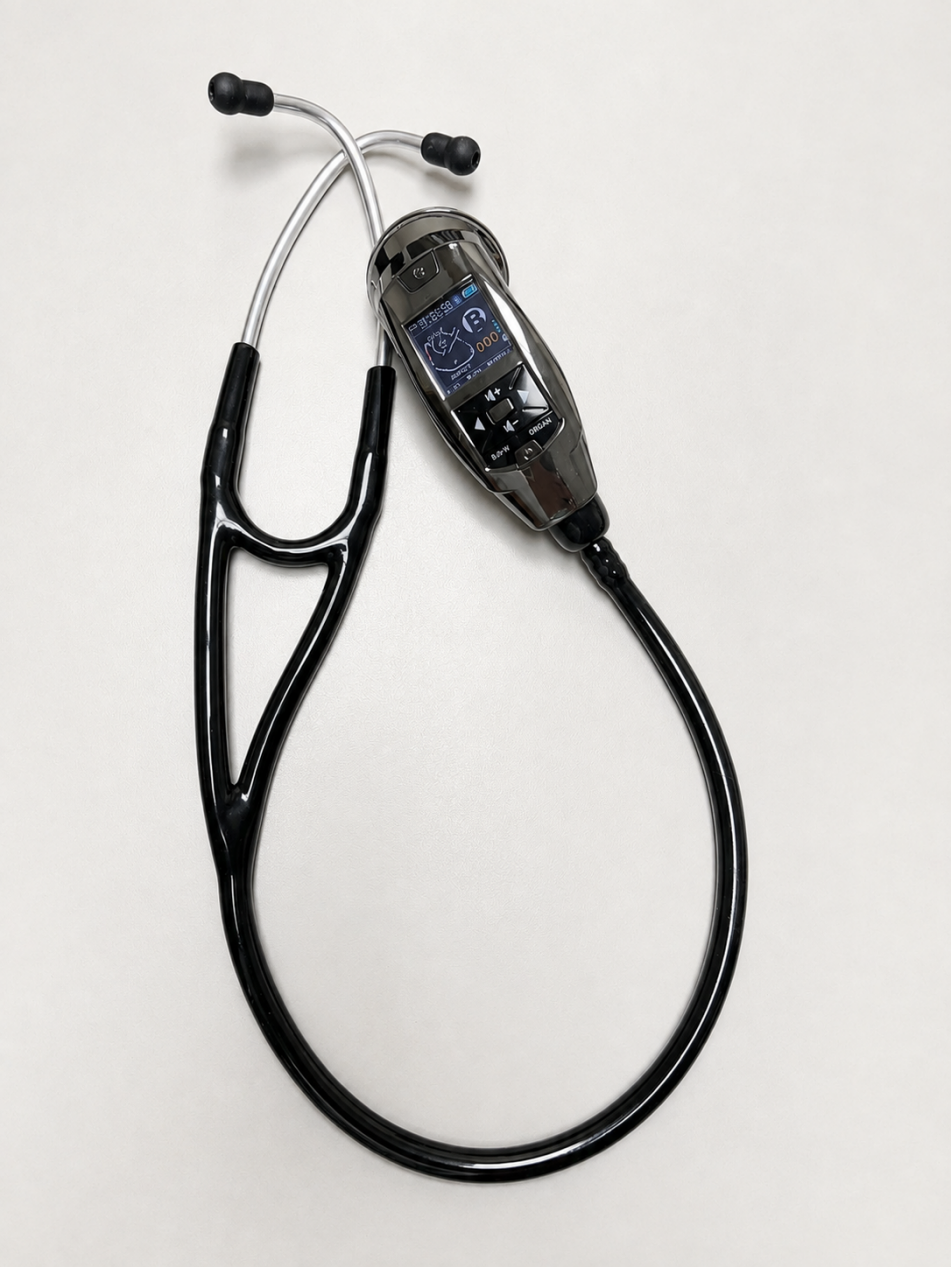
DS101 electronic stethoscope. Photograph of the DS101 electronic stethoscope (Chuang-Xin Medical Electronics) used for arteriovenous fistula acoustic monitoring. The device has a digital display with real-time waveform visualization and connects to a laptop computer via an adapter cable for recording and data transfer.

**Figure 2.**
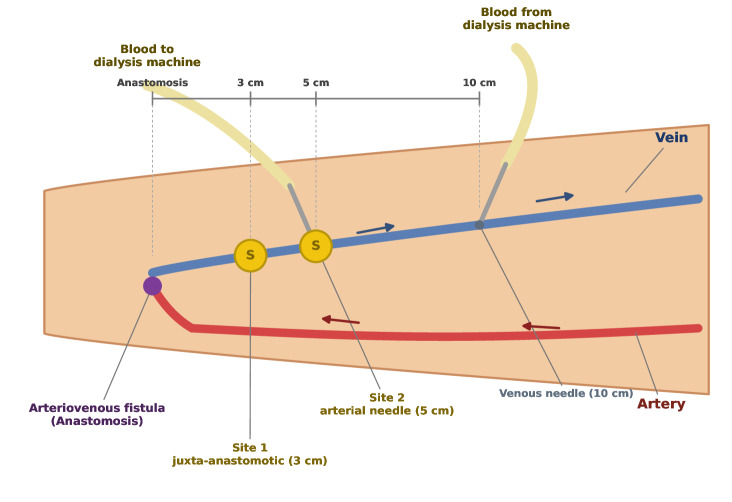
Electronic stethoscope measurement sites on AVF. Schematic of a radiocephalic arteriovenous fistula. The arterial limb (red) joins the venous outflow (blue) at the anastomosis (purple). Two standardized recording sites were used: Site 1, approximately 3 cm proximal to the anastomosis (juxta-anastomotic segment); and Site 2, the arterial needle insertion site (approximately 5 cm from the anastomosis, where blood is drawn to the dialysis machine). The venous return needle (approximately 10 cm; blood returning from the dialysis machine) is shown for orientation. Arrows indicate the direction of blood flow.

**Figure 3.**
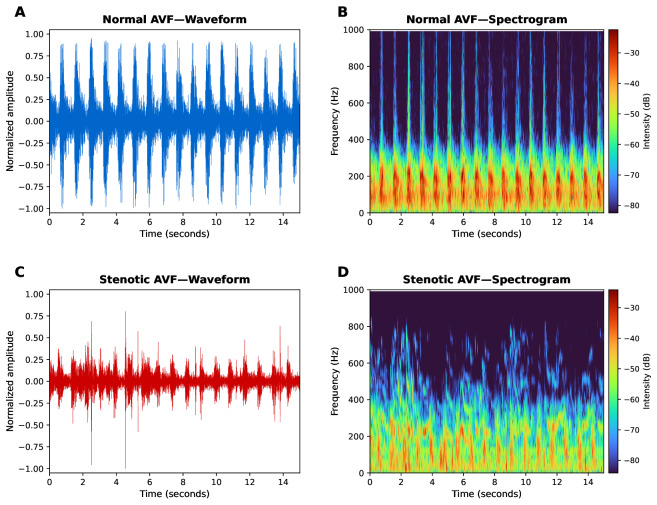
Acoustic characteristics of normal versus stenotic AVF. Representative waveforms (panels A and C) and short-time Fourier transform spectrograms (panels B and D) recorded with the DS101 electronic stethoscope and analyzed in Audacity (version 3.2.0; fast Fourier transform size 2048, Hann window). (A and B) Normal AVF (patient 1, site 2): a regular, pulsatile waveform with acoustic energy concentrated below approximately 400 Hz and rapid decay between beats. (C and D) Stenotic AVF (patient 27, site 2, before percutaneous transluminal angioplasty): a lower-amplitude, sustained, turbulent waveform with energy extending to 600-800 Hz. The color scale represents signal intensity from low (blue) to high (red). Group acoustic levels (segmented range mean, decibels relative to full scale): normal −24.95±1.21 (n=1373 recordings from nonstenotic patients); stenotic −29.76±0.41 (n=8 pre-PTA recordings from the single Doppler-confirmed patient; *P*<.001 by *t* test). Because all stenotic recordings derive from 1 patient, this comparison is descriptive of a single case and does not represent independent diagnostic performance. AVF: arteriovenous fistula.

The DS101 electronic stethoscope (Chuang-Xin Medical Electronics) was used for serial digital phonoangiographic monitoring of AVF sounds in this study (Figure 1). The device features a digital display showing real-time waveform visualization, multiple recording modes, and USB connectivity for data transfer to a laptop computer. Technical specifications include frequency response 20‐2000 Hz, digital sampling rate 44100 Hz, and dynamic range >70 dB. The handheld design with contact sensor enables standardized measurement at predetermined anatomical sites (Figure 2). All acoustic recordings were 20 seconds in duration and processed using Audacity software (version 3.2.0; Audacity Team) with STFT analysis. The signal processing pipeline consisted of four primary stages: (1) Waveform visualization: initial temporal inspection of sound amplitude patterns to ensure signal integrity (Figures 3A and 3C). (2) Spectrum analysis: transformation into the frequency domain to identify characteristic vascular bruit patterns. (3) STFT: conversion of the signals into a time-frequency representation, allowing for the observation of spectral changes over the recording duration. (4) Decibel calculation: amplitude values were converted to a logarithmic decibel scale using the standard reference formula: dB=20 × log₁₀ (amplitude/reference; Figures 3B and 3D).

### Additional Acquisition and Processing Details

AVF sounds were captured with a DS101 electronic stethoscope (Chuang-Xin Medical Electronics) connected to a laptop via an adapter cable, at 2 standardized sites—approximately 3 cm proximal to the anastomosis and at the arterial needle insertion site—for approximately 20 seconds per site and stored as 44.1-kHz mono audio. Each recording was analyzed in Audacity (version 3.2.0) by the STFT (fast Fourier transform size 2048; Hann window), producing 1025 frequency-bin magnitudes expressed in decibels relative to full scale (dBFS; 0 dBFS=maximum representable digital amplitude, so all values are ≤0). dBFS is a relative digital-level scale that is not referenced to the human hearing threshold, and the sign of a dBFS value does not by itself distinguish vascular sound from ambient noise; recordings were therefore obtained under standardized limb positioning with light, reproducible contact pressure by a single trained operator, and recordings degraded by motion or ambient noise were excluded (accounted for in the STROBE [Strengthening the Reporting of Observational Studies in Epidemiology] participant flow diagram). Band-pass filtering was not applied in the reported pipeline; each recording was summarized by a single decibel feature using the segmented-range-mean method (Results section). Each 20-second recording yielded a high-density dataset comprising 1025 discrete frequency-bin decibel values for subsequent analysis.

### Outcome Measures

The primary end point of this study was the occurrence of clinically significant AVF stenosis. Clinical stenosis was initially identified by an attending nephrologist through a standardized physical examination. Diagnostic criteria for clinical suspicion included acoustic abnormalities (conversion from a low-pitched continuous rumble to a high-pitched bruit), or detection of pulsatile flow or a significantly diminished thrill. Following clinical suspicion, the diagnosis was confirmed using color Doppler ultrasound, with stenosis defined as a diameter reduction of >50% at the target lesion. All confirmed cases subsequently underwent PTA as the standard interventional treatment to restore vascular patency. The secondary end point was the establishment of abnormal AVF acoustic threshold. This was achieved through a systematic statistical analysis of acoustic signals recorded following successful PTA. These “postintervention” sounds served as the baseline for normal vascular function. By comparing these baseline values with prestenotic signals, we established specific decibel cutoff values to objectively differentiate between physiological flow and pathological stenosis.

### Statistical Analysis

All analyses were exploratory and hypothesis-generating. Statistical analyses were performed using SPSS Statistics (version 26.0; IBM Corp), R (version 4.3; R Core Team) with the logistf package for penalized regression, and Microsoft Excel 2021 for data management. A 2-sided *P* value <.05 was considered statistically significant, with the caveat that all inferential results are exploratory. Because acoustic recordings were clustered within patients, they were not treated as independent observations; analyses were performed at the patient level using per-patient summaries. To account for the repeated-measures structure, we additionally fitted linear mixed-effects models with a random intercept for patient and quantified within-patient clustering using the intraclass correlation coefficient (ICC). The abnormal-sound threshold, defined a priori by a 3SD rule, was examined in a sensitivity analysis excluding the most influential patient, and its classification performance was assessed by leave-one-patient-out cross-validation (case-level sensitivity, recording-level specificity, and area under the receiver-operating-characteristic curve). Two distinct questions were addressed separately: as a proof-of-concept, we describe the single patient with duplex Doppler–confirmed stenosis (which predated monitoring) and the temporal relationship among Doppler findings, serial acoustic signals, and routine bedside recognition, with no modeling applied to this single event, and, in a cross-sectional analysis, we examined factors associated with a prespecified high-risk AVF phenotype (PTA within the preceding 3 years). Because only 9 patients met the high-risk definition, the number of events per variable precluded a conventional multivariable model; univariable associations were therefore assessed using the Fisher exact test (categorical variables) and independent *t* tests (continuous variables). Odds ratios (ORs) were estimated by Firth penalized (bias-reduced) logistic regression with profile-penalized 95% CIs, which yield finite estimates in the presence of complete separation; continuous predictors were additionally expressed as Firth ORs per unit increment (systolic BP per 10 mm Hg; prior PTA count per additional procedure). Differences in stenosis rates across the 4 dialyzer types were compared descriptively. Linear mixed-effects models and leave-one-patient-out cross-validation were performed in Python (version 3.10; Python Software Foundation) (statsmodels and scikit-learn). Reporting follows the STROBE guideline, and a participant flow diagram is provided.

To establish an objective baseline for detecting stenotic sounds without prior knowledge of clinical outcomes, a multistage empirical threshold determination process was used. The initial reference profile was derived using the 3SD method applied to a representative “normal” acoustic signature. The resulting threshold was derived in-sample and is therefore exploratory; its dependence on the single patient with Doppler-confirmed stenosis was examined in a sensitivity analysis, and its classification performance was evaluated by leave-one-patient-out cross-validation. External, prospective validation in an independent cohort is required before clinical use.

### Sample Size Justification

As this was a pilot study, the sample size was determined by the available patient population meeting the inclusion criteria (124 patients with potential AVF) and institutional time constraints. The final cohort of 29 patients yielded a dataset of 1462 acoustic measurements suitable for exploring acoustic thresholds. However, with only 9 high-risk patients, this cohort did not meet the recommended minimum of approximately 10 events per variable for multivariable logistic regression; accordingly, the risk-factor analysis is exploratory and was conducted using penalized and exact methods rather than a conventional multivariable model, and the early detection observation rests on a single illustrative case.

## Results

### Baseline Characteristics

A total of 29 patients completed the study protocol and were included in the final analysis. Table 1 summarizes the baseline characteristics of participants, stratified by AVF patency status. The stable patency group (n=20) was defined by the absence of PTA procedures within the preceding 3 years. Compared with the stable patency group, patients in the high-risk stenosis group (n=9) exhibited a significantly lower mean BMI (21.2 vs 24.8 kg/m²; *P*=.004) and a higher prevalence of diabetes (88.9% vs 30.0%; *P*=.005) and hypertension (100% vs 60.0%; *P*=.02). Furthermore, this high-risk group demonstrated higher mean systolic BP (153.7 vs 135.1 mm Hg; *P*=.02), more frequent use of FX dialyzers (77.8% vs 40.0%; *P*=.048), and a greater history of prior PTA procedures (3.44 vs 0.95; *P*=.002).

**Table 1.** Baseline patient characteristics (N=29).

Characteristics	Stable patency group (n=20)	High-risk stenosis group (n=9)	*P* value^a^
Demographics			
Age (years), mean (SD)	62.4 (14.2)	68.1 (10.8)	.29
Age ≥65 years, n (%)	8 (40)	6 (66.7)	.19
Female sex, n (%)	6 (30)	5 (55.6)	.19
BMI ≤25 kg/m², n (%)	10 (50)	9 (100)	*.006*
BMI (kg/m²), mean (SD)	24.8 (3.4)	21.2 (2.1)	*.004*
Comorbidities			
Diabetes mellitus, n (%)	6 (30)	8 (88.9)	*.005*
Hypertension, n (%)	12 (60)	9 (100)	*.02*
Dyslipidemia, n (%)	4 (20)	3 (33.3)	.44
Current smoker, n (%)	2 (10)	1 (11.1)	.93
AVF^b^ characteristics			
AVF location: forearm, n (%)	14 (70)	7 (77.8)	.67
AVF duration (months), mean (SD)	45.2 (38.6)	62.3 (44.1)	.30
Dialysis vintage (months), mean (SD)	48.7 (40.2)	65.8 (45.3)	.31
Dialysis vintage >6 months, n (%)	18 (90)	9 (100)	.34
Dialysis parameters			
Dialyzer size FX^c^, n (%)	8 (40)	7 (77.8)	*.048*
Blood flow rate (mL/min), mean (SD)	285 (24)	276 (28)	.37
Heparin initial dose (units), mean (SD)	1425 (312)	1350 (285)	.54
Heparin maintenance (units/hour), mean (SD)	850 (165)	825 (148)	.70
Blood pressure			
Mean systolic BP^d^ (mm Hg), mean (SD)	135.1 (16.4)	153.7 (22.9)	*.02*
Diastolic BP range (mm Hg), mean (SD)	14.2 (5.8)	16.8 (6.4)	.29
Laboratory values			
Albumin (g/dL), mean (SD)	3.9 (0.3)	3.7 (0.4)	.14
Low albumin (<3.5 g/dL), n (%)	3 (15)	3 (33.3)	.24
ESA^e^ use, n (%)	15 (75)	8 (88.9)	.39
Intervention history			
Prior PTA^f^ count, mean (SD)	0.95 (1.16)	3.44 (2.83)	*.002*

a Values in italics indicate statistical significance (*P*<.05).

b AVF: arteriovenous fistula.

c FX: Fresenius FX-series.

d BP: blood pressure.

e ESA: erythropoiesis-stimulating agents.

f PTA: percutaneous transluminal angioplasty.

### Risk Factor Analysis

Because only 9 patients met the high-risk definition, a conventional multivariable model was not statistically supportable and showed complete separation for hypertension and BMI. We therefore report exploratory univariable associations estimated with Firth penalized logistic regression and Fisher exact tests (Table 2). Diabetes mellitus (Firth OR 12.6, 95% CI 2.2‐138.1; Fisher exact *P*=.005), hypertension (OR 12.9, 95% CI 1.3‐31.7; *P*=.03), and BMI ≤25 kg/m² (OR 19.0, 95% CI 2.0‐2568; *P*=.01) were each associated with the high-risk phenotype; the wide confidence intervals reflect the small number of events. Penalization resolved the complete separation previously observed for hypertension and BMI and yielded finite estimates. On continuous Firth penalized regression, higher mean systolic BP (OR 1.53 per 10 mm Hg, 95% CI 1.04‐2.49; *P*=.03) and a greater number of prior PTAs (OR 1.74 per additional PTA, 95% CI 1.15‐3.40; *P*=.007) were each associated with the high-risk phenotype (Table 2). In contrast, the association with FX dialyzer use did not reach significance on exact testing (Firth OR 4.4, 95% CI 0.9‐28.5; Fisher exact *P*=.11) and is regarded as hypothesis-generating. All associations are exploratory and cannot be interpreted causally.

**Table 2.** Exploratory univariable associations with a high-risk AVF^a^ phenotype (Firth penalized logistic regression and Fisher exact tests)^b^.

Risk factor	High-risk (n=9)	Stable patency (n=20)	Firth OR^c^ (95% CI)	Fisher exact *P* value
Categorical, n (%)				
Diabetes mellitus	8 (88.9)	6 (30)	12.6 (2.2‐138.1)	.005
Hypertension	9 (100)	12 (60)	12.9 (1.3‐31.7)	.03
BMI ≤25 kg/m²	9 (100)	10 (50)	19.0 (2.0‐2568)	.01
FX^d^ dialyzer	7 (77.8)	8 (40)	4.4 (0.9‐28.5)	.11
Continuous, mean (SD)				
Mean systolic BP^e^, mm Hg^f^	153.7 (22.9)	135.1 (16.4)	1.53 per 10 mm Hg (1.04‐2.49)	.03
Prior PTA^g^ count^f^	3.44 (2.83)	0.95 (1.16)	1.74 per PTA (1.15‐3.40)	.007

a AVF: arteriovenous fistula.

b Odds ratios were estimated by Firth penalized (bias-reduced) logistic regression with profile-penalized 95% CIs; categorical *P* values are from the Fisher exact test. Penalization yields finite estimates despite the complete separation observed for hypertension and BMI under standard maximum-likelihood estimation.

c OR: odds ratio.

d FX: Fresenius FX-series.

e BP: blood pressure.

f Continuous variables were compared by *t *test (descriptive mean [SD]) and additionally expressed as Firth penalized odds ratios per unit increment (systolic BP per 10 mm Hg; prior PTA count per additional procedure). Wide confidence intervals reflect the small number of high-risk patients (9 events). All associations are exploratory and hypothesis-generating; they describe a prevalent high-risk phenotype (prior PTA within 3 years), not prospective incident stenosis.

g PTA: percutaneous transluminal angioplasty.

### Electronic Stethoscope Measurements

Throughout the 3-month prospective study period, a total of 1462 AVF acoustic recordings were collected and analyzed (Multimedia Appendix 2). Each participant contributed an average of 50.4 (SD 12.8) recordings. Measurements were equally distributed between 2 primary anatomical landmarks: the anastomosis (site 1, n=731) and the arterial needle cannulation site (site 2, n=731). Each 20-second recording yielded a high-density dataset comprising 1025 discrete decibel data points for subsequent analysis.

One participant (patient 27) had clinically significant stenosis that had already been confirmed by duplex Doppler ultrasonography before acoustic monitoring began and subsequently underwent PTA. To identify the most effective method for differentiating between stenotic and nonstenotic states, we compared the electronic stethoscope acoustic signals recorded before and after the PTA. We evaluated 3 distinct data aggregation approaches for the 1025 data points collected per recording.

- Method 1: Global mean. A simple arithmetic average of all 1025 decibel values was calculated. This approach failed to demonstrate a significant difference between the 2 periods (*P*=.322).
- Method 2: Negative value mean. Only negative decibel values were averaged. We note that the original rationale for this approach was not physically valid: because levels are expressed in dBFS, 0 dB denotes the maximum digital full-scale amplitude rather than the human hearing threshold, and the sign of a dBFS value does not distinguish vascular sound from environmental noise. This method also yielded nonsignificant results (*P*=.122).
- Method 3: Segmented range mean (selected method). Decibel values were stratified into 6 discrete decibel ranges (0 to −10, −10 to −20, −20 to −30, −30 to −40, −40 to −50, and −50 to −60 dB). The mean for each range was calculated independently, and the final representative value was defined as the average of these 6 range-specific means.

Statistical validation revealed that method 3 provided the highest discriminatory power, showing a highly significant difference between stenotic and nonstenotic periods (*P*<.001). Consequently, method 3 was adopted as the standardized aggregation protocol for all subsequent analyses. The differentiation between functional and stenotic AVF relies on the precise quantification of acoustic signatures captured via electronic auscultation. The reported acoustic parameter is the segmented-range-mean level of each recording, derived from the STFT frequency-bin magnitudes and expressed in dBFS (reference amplitude=1.0); it is a relative digital level, not a power spectral density, and is not referenced to the human hearing threshold. Because all stenotic recordings were obtained from the single Doppler-confirmed patient, the following comparison describes one case and its recordings are not independent observations. Normal, nonstenotic AVF sounds were characterized by a mean level of −24.95 (SD 1.21) dBFS. Spectrally, these signals are primarily distributed below approximately 400 Hz, exhibiting a rapid amplitude decay after the initial 0.3 seconds of the cardiac cycle. Visually, the resulting waveforms present a stable and consistent amplitude pattern. In contrast, stenotic AVF sounds showed a lower mean level of −29.76 (SD 0.41) dBFS; because these recordings all derive from 1 patient, this *P* value (<.001) should not be interpreted as independent evidence. The frequency spectrum in these cases extends higher, reaching 600‐800 Hz, with amplitude fluctuations that remain sustained beyond 0.3 seconds, creating an irregular and turbulent visual waveform.

To translate these acoustic differences into a reliable clinical diagnostic tool, a robust abnormal threshold was established using a 3SD statistical derivation method. Initial analysis focused on the post-PTA recordings of patient 27 (n=17), which served as a baseline for successful vascular restoration. These recordings yielded a mean of −25.48 (SD 1.42) dB, suggesting a theoretical 3SD range of −21.22 to −29.74 dB.

This preliminary threshold was then validated against a broader population of 1462 total recordings. The analysis identified 89 (6.1%) recordings falling below the −29.74 dB mark. Notably, 59.6% (n=53) of these outliers originated from 3 specific patients whose anatomical variation—anastomosis located at the arterial needle site—naturally altered the acoustic profile. The remaining 36 recordings represented either true pathological values or measurement artifacts.

To characterize this candidate threshold, the reference set was refined by excluding the 3 patients with anatomical variations, leaving 1373 recordings. The recalculated mean was −24.95 (SD 1.21) dBFS. Applying the 3SD rule to this refined set gave a candidate abnormal threshold of <−28.59 dBFS. Because this threshold was developed, refined, and evaluated within the same small dataset, it is reported only as an exploratory in-sample candidate that requires independent external validation. Within this dataset, it flagged 1 patient with Doppler-confirmed stenosis—the source of all abnormal recordings—while classifying 97.5% of the remaining recordings as normal; these figures describe internal consistency in a single case and must not be read as independent sensitivity or specificity. Because the 1462 recordings were clustered within patients, a linear mixed-effects model with a random intercept per patient was fitted; the ICC was 0.50, indicating that approximately half of the total variance in recording-level intensity was attributable to between-patient differences (recordings at the arterial needle site averaged 2.6 dB lower than at the anastomosis; *P*<.001). In leave-one-patient-out cross-validation of the 3SD rule, the single Doppler-confirmed stenosis case remained flagged when held out (case-level sensitivity 100%, ie, detection of 1 patient), with a recording-level specificity of 98.6% and an area under the curve of 0.90; because the entire positive class derives from 1 patient and recordings are clustered within patients, these recording-level figures are descriptive only and are not independent estimates of diagnostic accuracy. Excluding this patient shifted the threshold by only approximately 1.3 dB and left the abnormal-recording rate among the remaining patients essentially unchanged (1.43% vs 1.42%), indicating that specificity did not depend on that single patient.

### Illustrative Case: 1 Participant With Doppler-Confirmed Stenosis

On March 20, 2023 (day −30), the patient was enrolled in an electronic acoustic monitoring study. Initial recordings at site 2 (arterial needle site) yielded a mean acoustic level of −29.76 dBFS, below the reference level for healthy access (mean −24.95, SD 1.21 dBFS). Retrospective analysis confirmed that the electronic stethoscope identified the abnormal acoustic signature at the time of enrollment, reflecting a preclinical acoustic abnormality of at least 4 weeks. Importantly, duplex Doppler ultrasonography had already demonstrated stenosis on March 16 (day −34), 4 days before acoustic monitoring commenced; the abnormal acoustic signal was therefore apparent before routine nursing physical examination but not before imaging.

Between day −30 and day −2, serial monitoring showed consistent acoustic abnormalities. All site 2 recordings remained within a narrow, pathological range (−29.13 to −30.30 dB). The clinical diagnosis of stenosis (day 1; April 19, 2023) was triggered when nursing staff noted a high-pitched bruit and a diminished thrill during physical examination. This formal diagnosis occurred 30 days after the initial abnormal electronic recording, demonstrating a significant “detection lag” in traditional physical assessment protocols.

On day 5 (April 23, 2023), the patient underwent PTA. Angiography revealed an 89% stenosis located 3 cm proximal to the anastomosis (site 1). Technical success was achieved, with residual stenosis reduced to <30% postprocedure. One site 2 recording obtained on April 21, 2023—after the clinical diagnosis (April 19) but before PTA (April 23)—a periprocedural measurement was excluded from the pre- versus post-PTA comparison; the comparison therefore used 8 pre-PTA recordings (March 20 to April 17, 2023), whereas Multimedia Appendix 3 displays all 9 pre-PTA monitoring recordings. Following the PTA, acoustic monitoring from day 8 to day 59 showed immediate normalization of site 2 dB values, ranging from −24.43 to −25.90 dB (mean −25.22, SD 0,41 dB). Statistical analysis confirmed a highly significant difference between pre- and postintervention acoustic power (*P*<.001). A single outlier recorded on June 14 (−29.87 dB) was investigated and attributed to a measurement artifact rather than restenosis (Multimedia Appendix 3). Table 3 presents the statistical comparison of patient 27’s measurements.

The efficacy of the acoustic detection system was confirmed through significant improvements in sound intensity across both primary measurement sites following intervention. At site 1, which corresponds to the anastomosis, the mean acoustic intensity rose from −28.69 dB (pre-PTA) to −24.33 dB (post-PTA; *P*<.001). A similar trend was observed at site 2, located at the arterial needle site, where values increased from −29.76 dB (pre-PTA) to −25.48 dB (post-PTA; *P*<.001). Anatomical correlation during the PTA procedure confirmed the presence of stenosis at the site 1 location. Furthermore, the detection of abnormalities at site 2, likely resulting from retrograde flow disturbance caused by downstream narrowing, validates the clinical usefulness of using multiple measurement points to assess the patency of the AVF.

**Table 3.** Patient 27 acoustic measurements: pre-PTA^a^ vs post-PTA comparison.

Parameter	Pre-PTA (stenotic period)	Post-PTA (normal period)	*P* value^b^
Site 1 (3 cm proximal to anastomosis)			
Mean dB (SD)	−28.69 (1.98)	−24.33 (1.74)	*<.001*
Recordings analyzed, n	8	17	—^c^
Date range	March 20 to April 17, 2023	April 26 to June 16, 2023	—
Site 2 (arterial needle insertion)			
Mean dB (SD)	−29.76 (0.41)	−25.48 (1.42)	*<.001*
Recordings analyzed, n	8	17	—
Date range	March 20 to April 17, 2023	April 26 to June 16, 2023	—
Clinical events			
Doppler ultrasound stenosis detection	March 16, 2023	—	—
Clinical diagnosis by nursing staff	April 19, 2023	—	—
PTA procedure	April 23, 2023	—	—
Pre-PTA stenosis severity	89% diameter reduction	—	—
Post-PTA residual stenosis	—	<30%	—

a PTA: percutaneous transluminal angioplasty.

b *P* values in italics indicate statistical significance (*P*<.05).

c Not available.

### Dialyzer-Type Analysis

The distribution of dialyzer types among the 29 patients showed that FX was the most prevalent model (n=15, 51.7%), followed by FLX (6/29, 20.7%), F (5/29, 17.2%), and B3 (3/29, 10.3%). When examining stenosis rates by type, FX users had the highest proportion of patients meeting the high-risk definition (7/15, 46.7%), which was significantly greater than the rates observed for F (1/5, 20.0%), FLX (1/6, 16.7%), and B3 (0%) dialyzers (*χ*²_3_=7.912; *P*=.048).

To explore whether confounding by indication might contribute to these findings, a subgroup analysis of the high-risk stenosis cohort was performed, as shown in Table 4. This analysis showed no significant differences between dialyzer groups regarding age, BMI, diabetes prevalence, or dialysis vintage; however, the very small subgroup sizes limit this comparison and cannot exclude confounding. FX users trended toward lower mean acoustic intensity (mean −25.9, SD 2.8 dB) and a higher percentage of abnormal readings below the −28.59 dB threshold (12.4%), although the ANOVA for these acoustic trends did not reach statistical significance (*P*=.31). On exact testing, the FX association was not statistically significant (Fisher exact *P*=.11); given the small numbers, nonrandom dialyzer allocation, and likely residual confounding by indication, we regard it solely as a hypothesis-generating signal rather than a confirmed independent risk factor.

**Table 4.** Dialyzer-type comparison and association with AVF^a^ stenosis.

Dialyzer characteristic	F^b^	FX^c^	FLX^d^	B3^e^	*P* value^f^
Technical specifications					
Surface area (m²)	1.5	2.0	2.5	2.0	—^g^
Membrane material	Polysulfone	Polysulfone	Polysulfone	PMMA^h^	—
Blood flow capacity (mL/min), range	200‐300	250‐350	300‐450	250‐400	—
Typical treatment time (hours)	4	4	3.5‐4	4	—
Study population distribution					
Total patients, n (%)	5 (17.2)	15 (51.7)	6 (20.7)	3 (10.3)	—
High-risk stenosis group (n=9)					
Patients, n (%)	1 (11.1)	7 (77.8)	1 (11.1)	0 (0)	*.048*
Age (years), mean (SD)	72.0	67.4 (10.2)	70.0	—	.89
BMI, kg/m², mean (SD)	20.5	21.1 (2.0)	21.8	—	.81
Diabetes mellitus, n (%)	1 (100)	6 (85.7)	1 (100)	—	.82
Dialysis vintage (months), mean (SD)	58	68.3 (48.1)	62	—	.95
Blood flow (mL/min), mean (SD)	260	295 (28)	310	—	.42
Stable patency group (n=20)					
Patients, n (%)	4 (20)	8 (40)	5 (25)	3 (15)	—
Age (years), mean (SD)	64.5 (12.8)	61.8 (15.2)	60.4 (16.1)	65.3 (12.5)	.94
BMI (kg/m²), mean (SD)	25.2 (3.8)	24.6 (3.2)	24.9 (3.6)	24.5 (4.1)	1.00
Diabetes mellitus, n (%)	1 (25)	2 (25)	2 (40)	1 (33.3)	.91
Dialysis vintage (months), mean (SD)	42.5 (35.2)	51.8 (42.6)	46.2 (41.8)	48.0 (38.4)	.97
Mean blood flow (mL/min), mean (SD)	270 (22)	280 (25)	305 (30)	275 (20)	.16
Acoustic characteristics					
dB value (overall), mean (SD)	−24.8 (2.1)	−25.9 (2.8)	−24.6 (1.9)	−24.3 (1.6)	.31
Abnormal readings (<−28.59 dB), %	8.2	12.4	7.8	5.1	.16
Clinical outcomes					
Stenosis rate, %	20	46.7	16.7	0	*.048*
Prior PTA^i^ count (high-risk stenosis group), mean (SD)	2.0	3.7 (2.9)	3.0	—	.76
Odds ratio for stenosis (vs F)	1.0 (reference)	5.25	0.80	0	—
95% CI	—	1.02‐27.03	0.04‐16.28	—	—

a AVF: arteriovenous fistula.

b F: Fresenius F-series.

c FX: Fresenius FX-series.

d FLX: Fresenius FLX-series.

e B3: Baxter B3-series.

f *P* values in italics indicate statistical significance (*P*<.05).

g Not available.

h PMMA: polymethylmethacrylate.

i PTA: percutaneous transluminal angioplasty.

## Discussion

### Principal Findings

This prospective pilot study yielded 3 exploratory observations regarding AVF surveillance. First, in a single illustrative case, serial electronic stethoscope monitoring flagged an abnormal AVF signal approximately 1 month earlier than routine nursing physical assessment—although not earlier than duplex Doppler, which had already shown stenosis 4 days before monitoring began. Second, we derived a candidate acoustic cutoff (<−28.59 dB) that provided an objective, quantitative metric but was defined in-sample and remains to be externally validated. Third, in exploratory analyses, we identified candidate correlates of a high-risk AVF phenotype—diabetes mellitus, hypertension, BMI≤25, and higher mean systolic BP—together with a tentative, nonsignificant association with FX-class dialyzer use that we present only as hypothesis-generating.

The longitudinal data from our representative case (patient 27) highlight a critical “diagnostic gap” in current nephrology practice: significant stenosis remained clinically occult for at least 4 weeks before manifesting as physical symptoms. Unlike conventional physical examinations, which are inherently subjective and operator-dependent, electronic auscultation offers high reproducibility and quantitative trending. By transforming sound into discrete sound-level (dBFS) values, clinicians can move from “detecting failure” to “monitoring progression,” potentially bridging the detection lag that currently leads to acute thrombosis.

In exploratory univariable analysis, diabetes mellitus showed the strongest association with the high-risk phenotype (Firth OR 12.6), aligning with established literature on the role of diabetic vasculopathy and medial calcification in accelerated AVF failure [15-18]. Furthermore, the univariable associations of hypertension and elevated systolic BP are consistent with the “hemodynamic injury hypothesis,” suggesting that chronic high-pressure stress on the venous wall triggers neointimal hyperplasia [19]. The association between lower BMI and increased stenosis risk observed in our cohort aligns with the evidence provided by Tonelli et al [20], where higher BMI was linked to superior vascular access flow measurements by ultrasound dilution. Zheng et al [21] found that significant risk factors associated with postintervention patency of AVF and AVG included lower levels of serum albumin. In the dialysis population, lower BMI and serum albumin reflect the malnutrition-inflammation-atherosclerosis complex [22], often correlates with poor nutritional status and chronic inflammation, both of which are known drivers of vascular remodeling and access failure.

We observed a tentative association between FX dialyzer use and the high-risk phenotype (Firth OR 4.4), which did not reach significance on exact testing (Fisher exact *P*=.11) and must be interpreted with great caution, given the small numbers and nonrandom dialyzer allocation. Should it be confirmed in larger studies, several nonexclusive mechanisms could be hypothesized: (1) hemodynamic stress from prolonged moderate to high blood flow during FX sessions, (2) altered shear-stress distribution at the anastomosis related to membrane resistance and filtration fraction [23], and (3) membrane-related biocompatibility and inflammatory responses that could promote local neointimal growth [24]. We emphasize that these are speculative hypotheses and not established mechanisms.

These pilot findings are hypothesis-generating and are not sufficient to recommend changes to clinical practice. They do, however, motivate future evaluation of risk-stratified acoustic surveillance—for example, more frequent monitoring of patients with diabetes and a history of prior PTA—as a low-cost, noninvasive adjunct to physical examination that could prompt earlier confirmatory Doppler ultrasound. Whether such an approach improves access outcomes, and how it should be integrated with current KDOQI (Kidney Disease Outcomes Quality Initiative) guidance [4], should be tested in prospective studies.

### Limitations

This pilot study has important limitations. First, it is a single-center study of 29 patients over 3 months, and the single index patient had stenosis already confirmed by duplex Doppler before monitoring, so the case is illustrative of acoustic-clinical correspondence rather than of incident or early detection, and all abnormal recordings came from this 1 participant. Second, the risk-factor analysis is cross-sectional and uses a prevalent high-risk phenotype (prior PTA within 3 years) rather than prospective incident events; with only 9 high-risk patients, estimates are unstable, several predictors showed complete separation under standard modeling, and—despite penalized estimation—the associations remain exploratory and must not be interpreted causally. Third, the <−28.59 dBFS threshold was developed, refined, and evaluated within the same dataset; it is therefore only an exploratory in-sample candidate, and internal cross-validation cannot substitute for external validation. Fourth, the 1462 recordings were clustered within patients (mixed-effects ICC 0.50); although we accounted for this with patient-level analyses and a random-intercept model, residual measurement variability (contact pressure, ambient noise, dialyzer, or needle artifact) cannot be excluded despite patient-level analysis. Fifth, the FX-dialyzer signal was not significant on exact testing and is likely subject to confounding by indication, as dialyzer selection is clinically driven.

To address these limitations, future research should prioritize the following: (1) Large-scale multicenter trials: validating the established decibel threshold and risk factors in prospective studies with cohorts exceeding 200 patients. (2) Automated diagnostic systems: integrating machine learning algorithms to provide real-time, objective risk assessments at the bedside. (3) Hemodynamic and mechanistic studies: using computational fluid dynamics modeling and randomized dialyzer protocols to clarify the precise impact of FX-class membranes on vascular shear stress and neointimal hyperplasia. (4) Biomarker integration: investigating the correlation between acoustic spectral signatures and molecular biomarkers of vascular inflammation to better understand the pathophysiology of AVF failure.

### Conclusions

This prospective pilot study suggests that quantitative electronic acoustic monitoring is feasible and, in a single illustrative case, the abnormal acoustic signal in 1 patient with Doppler-confirmed stenosis was recognized before routine nursing assessment, though not before imaging. We derived a candidate acoustic threshold that provides an objective metric but requires external validation. Exploratory analyses identified candidate correlates of a high-risk AVF phenotype, including diabetes mellitus, hypertension, low BMI, elevated systolic BP, and prior PTA; a tentative association with FX-class dialyzer use was not significant on exact testing and is hypothesis-generating. These findings should be confirmed in larger, multicenter, and externally validated studies before informing surveillance or dialyzer selection strategies.

## Supplementary material

10.2196/97202 Multimedia Appendix 1 Participant flow (STROBE [Strengthening the Reporting of Observational Studies in Epidemiology]) diagram. Flow of participants through the study. Of 156 patients at the hemodialysis center, 124 patients with a native AVF were assessed for eligibility; 32 were excluded for nonnative access (arteriovenous graft, n=21; tunneled cuffed catheter, n=11) and 94 were not enrolled (did not meet all inclusion criteria, or declined or were unable to give consent). Thirty patients were enrolled, 1 withdrew during follow-up (personal reasons, May 22, 2023), and 29 completed the 3-month protocol and were included in the analysis (1462 acoustic recordings; site 1 [anastomosis], n=731; site 2 [arterial needle], n=731). The single stenosis event was confirmed by duplex Doppler before acoustic monitoring and was not an incident (newly developing) stenosis. Single-center cohort, National Taiwan University Hospital Yunlin Branch, March-June 2023. AVF: arteriovenous fistula; HD: hemodialysis; PTA: percutaneous transluminal angioplasty.

10.2196/97202 Multimedia Appendix 2 Per-recording acoustic dataset (all recordings). Spreadsheet (Microsoft Excel; XLSX) containing all 1464 AVF sound recordings from the 30 enrolled patients (March-June 2023). Recordings were captured with a DS101 electronic stethoscope (Chuang-Xin Medical Electronics) at 2 standardized sites (approximately 3 cm proximal to the anastomosis and the arterial needle insertion site), approximately 20 seconds each, and stored as 44.1-kHz mono audio. The “Recordings” sheet provides 1 row per recording with patient identifier, site, date, duration, and computed acoustic features (level in dBFS, peak level, dominant frequency, spectral centroid, and band-power fractions for the <200, 200-600, 600-800, and 800-1500 Hz bands); the “Patient_summary” sheet gives per-patient aggregates; and a “README” sheet defines every column and the processing method. In the study’s original pipeline, each recording was analyzed in Audacity (version 3.2.0) by the short-time Fourier transform (fast Fourier transform size 2048, Hann window), producing 1025 frequency-bin magnitudes expressed in dBFS (0 dBFS = maximum digital amplitude, so all values are ≤0); the per-recording value was the segmented-range-mean of these magnitudes, grouped into six 10-dB ranges from 0 to −60 dB and then averaged across the 6 range means. The abnormal-sound threshold (<−28.59 dBFS) was derived from this feature by a 3SD rule (the single Doppler-confirmed patient’s post-PTA recordings gave a stenotic characteristic of <−29.07 dBFS; applying the 3SD rule to all recordings above −29.07 dBFS yielded the −28.59 dBFS threshold). The dBFS values contained in this spreadsheet are a separate, reproducible broadband measure computed directly from the raw audio for transparency and are therefore on a different absolute scale from the study’s Audacity segmented-range-mean values. AVF: arteriovenous fistula; dBFS: decibels relative to full scale; PTA: percutaneous transluminal angioplasty.

10.2196/97202 Multimedia Appendix 3 Index case (patient 27): site 2 acoustic level across follow-up. Segmented-range-mean acoustic level (dBFS) at the arterial needle site (site 2) for the single Doppler-confirmed stenosis case across 26 recording visits (March 20 to June 16, 2023). The per-recording level was computed from the short-time Fourier transform (Audacity version 3.2.0; fast Fourier transform size 2048, Hann window) as the segmented-range-mean of the 1025 frequency-bin magnitudes, grouped into six 10-dB ranges spanning 0 to −60 dB. The plot shows all 9 pre-PTA monitoring recordings; the pre- versus post-PTA statistical comparison used 8 pre-PTA recordings, excluding the April 21 recording, which was obtained after the clinical diagnosis (April 19) but before PTA (April 23) and was therefore periprocedural. PTA was performed on April 23, 2023 (dotted vertical line). The dashed horizontal line marks the exploratory in-sample abnormal sound threshold (<−28.59 dBFS). All pre-PTA recordings fall below the threshold, and 16 of 17 post-PTA recordings are above it; the single value below threshold after PTA (June 14) reflects a probe position artifact rather than vascular dysfunction. These are descriptive single-case data and do not constitute an independent estimate of diagnostic accuracy. dBFS: decibels relative to full scale; PTA: percutaneous transluminal angioplasty.

## Abbreviations

AVF: arteriovenous fistula
AVG: arteriovenous graft
BP: blood pressure
dB: decibel
dBFS: decibels relative to full scale
ESRD: end-stage renal disease
FLX: Fresenius FLX-series
FX: Fresenius FX-series
HD: hemodialysis
ICC: intraclass correlation coefficient
IRB: institutional review board
KDOQI: Kidney Disease Outcomes Quality Initiative
NTUH: National Taiwan University Hospital
OR: odds ratio
PTA: percutaneous transluminal angioplasty
STFT: short-time Fourier transform
STROBE: Strengthening the Reporting of Observational Studies in Epidemiology
