# Supplementary material for: Risk Factors of Arteriovenous Fistula Stenosis in Hemodialysis Patients Using Electronic Stethoscope Monitoring: Prospective Pilot Cohort Study
Source: JMIR Form Res. 2026 Aug 18;10:e97202. doi: 10.2196/97202 (PMC13485153; doi:10.2196/97202)

Multimedia Appendix 1

Participant Flow (STROBE) Diagram

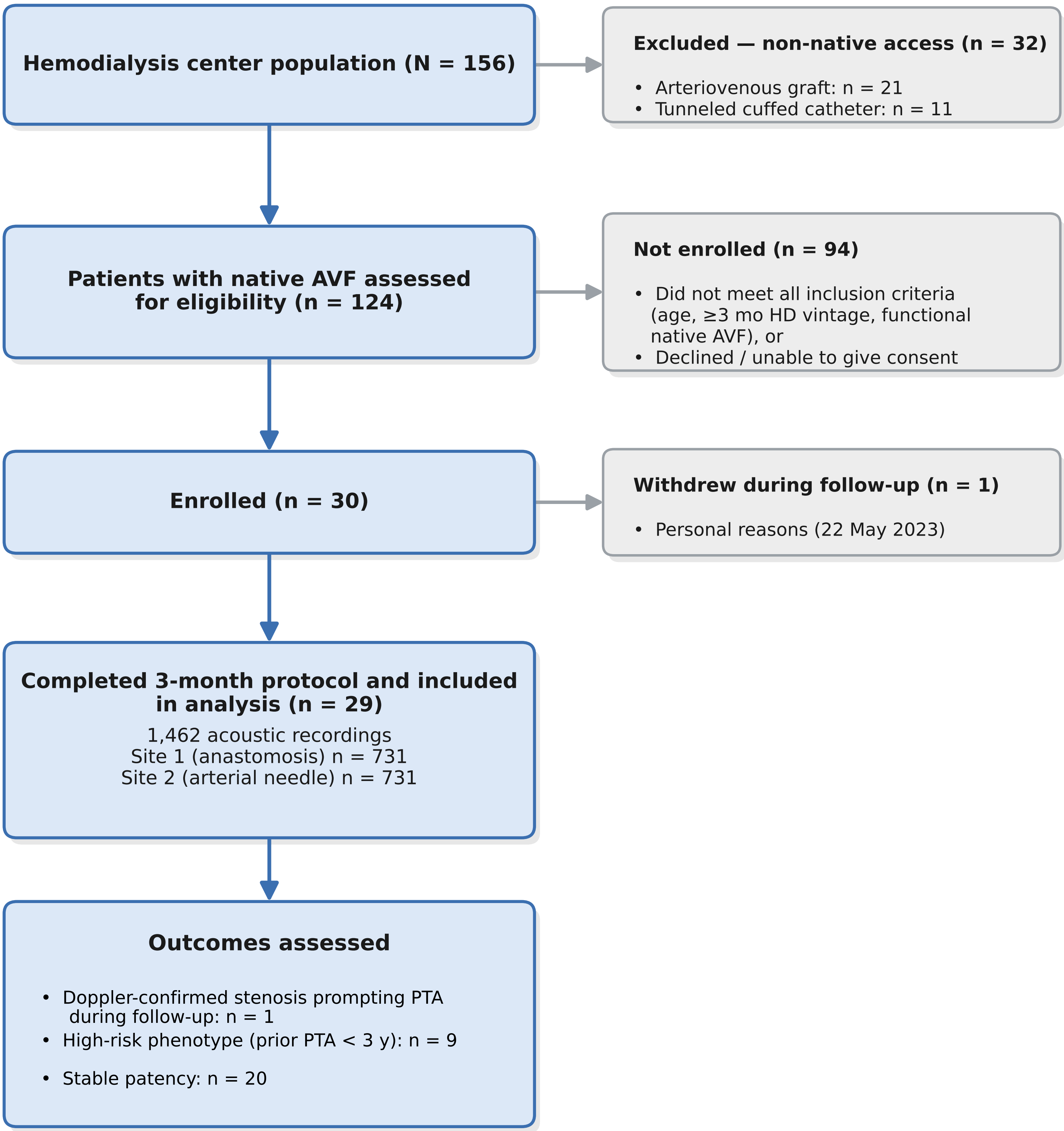

Supplement: Supplementary file 1 [file formative-v10-e97202-s001.pdf]
